# Give it a rest: a systematic review with Bayesian meta-analysis on the effect of inter-set rest interval duration on muscle hypertrophy

**DOI:** 10.3389/fspor.2024.1429789

**Published:** 2024-08-14

**Authors:** Alec Singer, Milo Wolf, Leonardo Generoso, Elizabeth Arias, Kenneth Delcastillo, Edwin Echevarria, Amaris Martinez, Patroklos Androulakis Korakakis, Martin C. Refalo, Paul A. Swinton, Brad J. Schoenfeld

**Affiliations:** ^1^ Department of Exercise Science and Recreation, Applied Muscle Development Lab, CUNY Lehman College, Bronx, NY, United States; ^2^ Institute for Physical Activity and Nutrition (IPAN), School of Exercise and Nutrition Sciences, Deakin University, Geelong, VIC, Australia; ^3^ Department of Sport and Exercise, School of Health Sciences, Robert Gordon University, Aberdeen, United Kingdom

**Keywords:** rest period, recovery interval, muscle growth, muscle development, muscle thickness, muscle cross-sectional area

## Abstract

We systematically searched the literature for studies with a randomized design that compared different inter-set rest interval durations for estimates of pre-/post-study changes in lean/muscle mass in healthy adults while controlling all other training variables. Bayesian meta-analyses on non-controlled effect sizes using hierarchical models of all 19 measurements (thigh: 10; arm: 6; whole body: 3) from 9 studies meeting inclusion criteria analyses showed substantial overlap of standardized mean differences across the different inter-set rest periods [binary: short: 0.48 (95%CrI: 0.19–0.81), longer: 0.56 (95%CrI: 0.24–0.86); Four categories: short: 0.47 (95%CrI: 0.19–0.80), intermediate: 0.65 (95%CrI: 0.18–1.1), long: 0.55 (95%CrI: 0.15–0.90), very long: 0.50 (95%CrI: 0.14–0.89)], with substantial heterogeneity in results. Univariate and multivariate pairwise meta-analyses of controlled binary (short vs. longer) effect sizes showed similar results for the arm and thigh with central estimates tending to favor longer rest periods [arm: 0.13 (95%CrI: −0.27 to 0.51); thigh: 0.17 (95%CrI: −0.13 to 0.43)]. In contrast, central estimates closer to zero but marginally favoring shorter rest periods were estimated for the whole body [whole body: −0.08 (95%CrI: −0.45 to 0.29)]. Subanalysis of set end-point data indicated that training to failure or stopping short of failure did not meaningfully influence the interaction between rest interval duration and muscle hypertrophy. In conclusion, results suggest a small hypertrophic benefit to employing inter-set rest interval durations >60 s, perhaps mediated by reductions in volume load. However, our analysis did not detect appreciable differences in hypertrophy when resting >90 s between sets, consistent with evidence that detrimental effects on volume load tend to plateau beyond this time-frame.

**Systematic Review Registration:** OSF, https://doi.org/10.17605/OSF.IO/YWEVC.

## Introduction

It has been proposed that the manipulation of resistance training (RT) program variables can help to optimize skeletal muscle hypertrophy (1). However, because of the onerous time commitment involved in conducting directly supervised longitudinal RT protocols, most research on the effects of manipulation of program variables have involved relatively small sample sizes. Thus, meta-analytic techniques that pool and explore the results of all relevant studies on a given topic can provide additional insights on the topic by quantifying the magnitude of effects, which may help to guide prescription. To date, relatively recent meta-analyses have investigated the effect of manipulating a variety of RT program variables on muscle hypertrophy outcomes including load (2), volume (3), frequency (4), and proximity to failure (5), furthering our understanding of their practical implications.

The rest interval, operationally defined herein as the duration between sets during RT, is thought to be an important variable that has implications for exercise prescription (6). The National Strength and Conditioning Association recommends relatively short rest periods (30–90 s) to optimize muscle hypertrophy (7). This is largely based on acute research showing that short rest periods enhance the post-exercise hormonal response to RT, which has been theorized to promote muscular adaptations (8). However, emerging research suggests that transient post-exercise hormonal elevations may not play an important role in eliciting muscle hypertrophy (9, 10), which calls into question the benefit of short rest intervals for optimizing muscle development. Moreover, there is an inverse relationship between rest interval duration and the magnitude of load lifted in subsequent sets, whereby shorter rest periods necessitate larger reductions in load to complete a given number of repetitions compared to longer rest periods (11, 12). Considering that mechanical tension is a primary mechanism for promoting RT-induced hypertrophy (13), such reductions in volume load may actually compromise muscular adaptations. Indeed, McKendry et al. (14) reported that short rest intervals (1 min) blunted the myofibrillar protein synthetic response to RT compared to longer rest intervals (5 min) despite higher acute testosterone elevations in the short-rest condition; predictably, volume load decreased to a greater extent with shorter rest.

Longitudinal research investigating the influence of rest intervals on muscle hypertrophy has been largely equivocal. A systematic review by Grgic et al. (15) concluded that both short and long inter-set rest periods are viable options for untrained individuals seeking to optimize hypertrophy, but that longer durations may be advantageous for those with previous RT experience. It should be noted that this review was published in 2017 and additional research has been conducted on the topic since that time. Moreover, no study to date has endeavored to quantify the magnitude of effect between different rest interval conditions to determine if differences may be practically meaningful for RT prescription. Therefore, the purpose of this study was to systematically review the literature and perform a Bayesian meta-analysis of the existing data on the effects of rest interval duration during RT on measures of muscle hypertrophy.

## Materials and methods

We conducted this review in accordance with the guidelines of the “Preferred Reporting Items for Systematic Reviews and Meta-Analyses” (PRISMA). The study was preregistered on the Open Science Framework (https://osf.io/ywevc).

### Literature search strategy

To identify relevant studies for the topic, we conducted a comprehensive search of the PubMed/MEDLINE, Scopus, and Web of Science databases using the following Boolean search syntax: (“rest interval” OR “inter-set rest” OR “interset rest” OR “rest period*” OR “rest between sets” OR “resting interval” OR “resting period” OR “recovery interval”) AND (“resistance training” OR “resistance exercise” OR “weight lifting” OR “weightlifting” OR “strength exercise” OR “strength training” OR “strengthening” OR “resistive exercise” OR “resistive training”) AND (“muscle hypertrophy” OR “muscular hypertrophy” OR “muscle mass” OR “lean body mass” OR “fat-free mass” OR “fat free mass” OR “muscle fiber” OR “muscle size” OR “muscle fibre” OR “muscle thickness” OR “cross-sectional area” OR “computed tomography” OR “magnetic resonance imaging” OR “ultrasound” OR “DXA” OR “DEXA” OR “bioelectrical impedance analysis”)*.* As previously described (16), we also screened the reference lists of articles retrieved and applicable review papers, as well as tapped into the authors' personal knowledge of the topic, to uncover any additional studies that might meet inclusion criteria (17). Moreover, we performed secondary “forward” and “backward” searches for citations of included studies in Google Scholar.

As previously described, the search process was conducted separately by 3 researchers (LG, AS and MR). Initially, we screened all titles and abstracts to uncover studies that might meet inclusion/exclusion criteria using online software (https://www.rayyan.ai/). If a paper was deemed potentially relevant, we scrutinized the full text to determine whether it warranted inclusion. Any disputes that could not be resolved by the search team were settled by a fourth researcher (BJS). The search was finalized in March 2024.

### Inclusion criteria

We included studies that satisfied the following criteria: (a) had a randomized design (either within- or between-group design) and compared different inter-set rest interval durations for estimates of pre-/post-study changes in lean/muscle mass using a validated measure (dual-energy x-ray absorptiometry [DXA], bioelectrical impedance analysis, magnetic resonance imaging [MRI], computerized tomography [CT], ultrasound, muscle biopsy or limb circumference measurement) in healthy adults (≥18 years of age) of any RT experience while controlling all other training variables (in the case of volume, this represented either sets per muscle per session or volume load per session [i.e., sets × repetitions × load]^1^; (b) involved at least 2 RT sessions per week for a duration of at least 4 weeks; (c) published in a peer-reviewed English language journal or on a preprint server. We excluded studies that (a) included participants with co-morbidities that might impair the hypertrophic response to RT (musculoskeletal disease/injury/cardiovascular impairments); (b) employed unequal dietary supplement provision (i.e., one group received a given supplement and the other received an alternative supplement/placebo).

### Data extraction

Three researchers (KD, EA and MW) independently extracted and coded the following data for each included study: Author name(s), title and year of publication, sample size, participant characteristics (i.e., sex, training status, age), description of the training intervention (duration, volume, frequency, modality), nutrition controlled (yes/no), method for lean/muscle mass assessment (i.e., DXA, MRI, CT, ultrasound, biopsy, circumference measurement), and mean pre- and post-study values for lean/muscle mass with corresponding standard deviations. In cases where rest periods fluctuated over time, we averaged values to report a mean. In cases where measures of changes in lean/muscle mass were not reported, we attempted to contact the corresponding author(s) to obtain the data as previously described (16). If unattainable, we extracted the data from graphs (when available) via online software (https://automeris.io/WebPlotDigitizer/). To account for the possibility of coder drift, a third researcher (AS) recoded 30% of the studies, which were randomly selected for assessment (18). Per case agreement was determined by dividing the number of variables coded the same by the total number of variables. Acceptance required a mean agreement of 0.90. Any discrepancies in the extracted data were resolved through discussion and mutual consensus of the coders.

### Methodological quality

The methodological quality of the included studies was assessed using the “Standards Method for Assessment of Resistance Training in Longitudinal Designs” (SMART-LD) scale (16). The SMART-LD tool consists of 20 questions that address a combination of study bias and reporting quality as follows: general (items 1–2); participants (items 3–7), training program (items 8–11), outcomes (items 12–16), and statistical analyses (17–20). Each item in the checklist is given 1 point if the criterion is sufficiently displayed or 0 points if the criterion is insufficiently displayed. The values of all questions are summed, with the final total used to classify studies as follows: “good quality” (16–20 points); “fair quality” (12–15 points); or “poor quality” (≤11). Three reviewers (EE, AM and PAK) independently rated each study using the SMART-LD tool; any disputes were resolved by majority consensus. We included all data irrespective of the study rating.

### Statistical analyses

All meta-analyses were conducted within a Bayesian framework enabling the results to be interpreted more intuitively compared to a standard frequentist approach through use of probabilistic statements regarding parameters of interest (19). A Bayesian framework avoids dichotomous interpretations of meta-analytic results regarding the presence or absence of an effect (e.g., with *p* values), and instead places greater emphasis on describing the most likely values for the average effect (19) while addressing practical questions such as which inter-set rest interval duration is likely to create the greatest muscle hypertrophy. To facilitate comparisons across the inter-set rest interval spectrum, durations were categorized using two sets of cut-points. The first was a binary categorization of short (duration ≤ 60 s) and longer (duration > 60 s), and the second comprised four categories (short: duration ≤ 60 s; intermediate: 60 s < duration < 120 s; long: 120 s ≤ duration < 180 s; and very long: duration ≥ 180 s). These cutoffs are based on the general rest interval durations used across studies. Due to the use of different measurement technologies, effect sizes were quantified by using standardized mean differences (SMDs). To account for the small sample sizes generally used in strength and conditioning, a bias correction was applied (20). The primary measure for this meta-analysis was controlled magnitude-based SMDs obtained by subtracting the baseline change of one inter-set rest interval category from another and dividing by the pre-intervention pooled standard deviation (20). To assess the overall effectiveness of the interventions included, initial analyses were conducted using non-controlled SMDs (21). Interpretation of the magnitude of effect sizes was facilitated by comparison to small, medium, and large thresholds developed for strength and conditioning outcomes (22).

Three-level hierarchical models were used with inter-set rest interval included as a categorical variable to summarize the results using non-controlled SMDs. Pairwise (direct comparisons only) and network (direct and indirect comparisons) meta-analysis approaches were then used with controlled SMDs to compare across the binary and four category representations, respectively. Univariate analyses separated by measurement site (whole body, thigh, or arm) were also conducted. For the direct comparison, multivariate analysis was also conducted allowing for correlations between measurement sites. Network meta-analyses are becoming increasingly common in evidence synthesis and are most used to compare qualitatively different treatments where individual studies are unlikely to directly compare all levels (23). The technique calculates pairwise effect sizes from studies comparing two levels (direct evidence) and generates indirect evidence comparing other levels through a common comparator (23). To summarize potential differences in hypertrophy across all inter-set rest interval categories in a network, the Surface Under the Cumulative Ranking curve (SUCRA (24); was used. For each category a SUCRA value expressed as a percentage was calculated representing the likelihood that muscle hypertrophy was highest or among the highest relative to other categories. Where applicable, we reported probabilities as *p*-values representing the proportion of the distribution that exceeded zero.

Informative priors were used for all models. For the hierarchical meta-regressions, the mean pre to post intervention change included an informative prior obtained from a large meta-analysis of strength and conditioning outcomes expressed in terms of SMDs (22). For controlled effect sizes, similar research in strength and conditioning conducted with comparative effect sizes was used (25). For the between-studies standard deviation, informative priors were based on an analysis of the predictive distributions generated from a large number of previous meta-analyses (26). It is a common limitation in meta-analyses using SMDs from intervention change scores to use a fixed value for the pre- to post-study correlation (e.g., a value of 0.7) not based on any empirical data (27). To account for this limitation, the sampling error for each study was estimated using an informative uniform prior with lower bound based on the sampling error calculated with a correlation of 0.9 and the upper bound based on the sampling error calculated with a correlation of 0.5. All analyses were performed in R, using the R2OpenBUGS package (28) for Bayesian sampling.

To improve accuracy, transparency and replication in the analyses, the WAMBS-checklist (When to worry and how to Avoid Misuse of Bayesian Statistics) was used and incorporated sensitivity analyses that included non-informative priors (29). Documentation for the WAMBS-checklist is provided in the supplementary files along with other diagnostics for primary analyses (including funnel plot and transitivity check for distribution of study characteristics across treatment comparisons in network). Consistency analyses were not conducted on networks due to insufficient data and a lack of loops in the networks.

## Results

**Figure 1:**
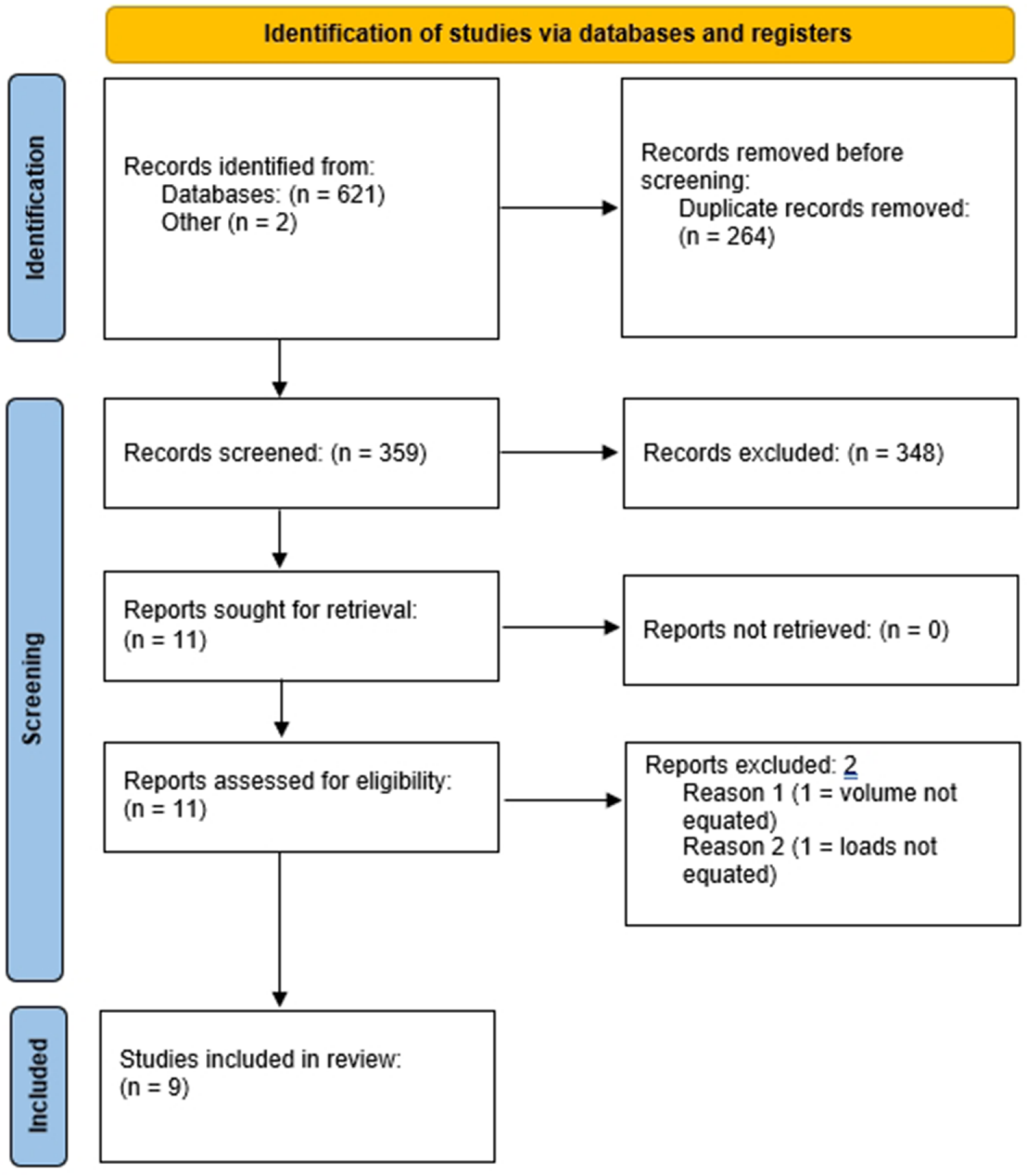
PRISMA flow chart of the search process.

We initially screened 359 studies and identified 11 that potentially met inclusion criteria. After reviewing the full texts of these studies, 2 studies were excluded: one because neither set volume nor volume load was equated between conditions (30) and the other because the loading range was not equated in the initial set of the given exercise(s) (31). Figure 1 provides a flow chart of the search process.

### Study characteristics

Eight studies employed young participants (18–35 years of age) (32–39) and 1 employed older participants (>65 years of age) (40). Six studies employed untrained participants (32–36, 40) and 3 studies employed resistance-trained participants (37–39). Six studies employed male participants (32, 33, 37–40), 1 study employed female participants (36), 1 study employed both male and female participants (35), and 1 study did not specify the sex of participants (34). Three studies assessed total body measures of hypertrophy (32, 33, 40), 5 studies assessed upper body measures of hypertrophy (biceps brachii and triceps brachii) (33, 34, 37–39), and 7 studies assessed lower body measures of hypertrophy (quadriceps femoris and total thigh) (33–39). The duration of the included studies ranged from 5 to 10 weeks. Table 1 provides a descriptive overview of each study's methodological design.

**Table 1:** Summary of the methods of included studies.

Study	Sample	Design	Exercises	RT protocol	Hypertrophy measure	Duration
Buresh et al. (33)	12 young, untrained men	Parallel group random assignment to 1 of 2 groups: (1) 60 s RI; (2) 150 s RI	Squat, leg curl, leg extensions, standing heel raise, seated dumbbell press, dumbbell lateral raises, rear delts on pec-deck, abdominal crunches, lying leg raises, pull-downs, machine rows, machine bench press, pec flies, incline dumbbell curls, machine biceps curls, dumbbell kickbacks	TB protocol performed 2 d/wk consisting of 2–3 sets of 10 repetitions per exercise	- Hydrodensitometry: FFM - Skinfold and CIR: CSA of arm and thigh	10 wks
de Souza et al. (37)	20 young, resistance-trained men	Parallel group random assignment to 1 of 2 groups: (1) 120 s RI; (2) RI decreasing from 120 s to 30 s (mean RI = ∼80 s)	Bench press, incline bench press, wide grip lat pulldown, leg extension, leg curl machine, front military press, dumbbell shoulder lateral raises, barbell curls, triceps pushdown, barbell lying triceps extension, abdominal crunches	TB protocol performed 6 d/wk consisting of 3–4 sets of 8–12 repetitions per exercise	- MRI: CSA of arm and thigh	8 wks
Fink et al. (31)	21 young, untrained individuals	Parallel group random assignment to 1 of 2 groups: (1) 30 s RI; (2) 150 s RI	Barbell curl, preacher curl, hammer curl, close grip bench press, French press, dumbbell extension	4 sets of squats and bench performed 2 d/wk at 40% 1RM	- MRI: CSA of triceps brachii and thigh	8 wks
Hill-Haas et al. (36)	18 young, untrained women	Parallel group random assignment to 1 of 2 groups: (1) 20 s RI; (2) 80 s RI	Parallel squats, bench step-ups with dumbbells, leg press (seated), dumbbell lunge, knee extensions, leg curls, bench press, seated rows, lat pull downs, dumbbell shoulder press. abdominal crunches	TB protocol performed 3 d/wk consisting of 2–5 sets of 15–20 repetitions per exercise	- CIR: thigh	5 wks
Longo et al. (35)	28 young, untrained men and women	Within-participant random assignment of legs to 1 of 4 conditions: (1) 60 s RI; (2) 180 s RI; (3) 60 s RI with VL equated to long RI; (4) 180 s RI with VL equated to short RI	Unilateral inclined leg press	3 sets of leg press performed 2 d/wk at 80% 1RM	- MRI: CSA of quadriceps femoris	10 wks
Piirainen et al. (32)	21 young, untrained men	Parallel group random assignment to 1 of 2 groups: (1) 55 s RI; (2) 120 s RI	Leg press, plantar flexion, bench press, elbow extension, shoulder press, low back, abdominal, knee extension, knee flexion, rowing, cable pulldown, upright row, back, trunk rotation	TB protocol performed 3 d/wk consisting of 3 sets of 10–20 repetitions per exercise	- BIA: FFM	7 wks
Schoenfeld et al. (39)	21 young, resistance-trained men	Parallel group random assignment to 1 of 2 groups: (1) 60 s RI; (2) 180 s RI	Barbell back squat, plate-loaded leg press, plate-loaded leg extension, flat barbell press, seated barbell military press, wide-grip plate-loaded lateral pulldown, plate-loaded seated cable row	TB protocol performed 3 d/wk consisting of 3 sets of 8–12 repetitions per exercise	- US: MT of biceps brachii, triceps brachii, quadriceps femoris	8 wks
Souza-Junior et al. (38)	22 young, resistance-trained men	Parallel group random assignment to 1 of 2 groups: (1) 120 s RI; (2) RI decreasing from 120 s to 30 s (mean RI = ∼80 s)	Bench press, incline bench press, wide grip lat pulldown, machine seated row, back squat, leg extension, leg curl machine, front military press, dumbbell shoulder lateral raises, barbell curls, alternating biceps curl with dumbbells, triceps pushdown, barbell lying triceps extension, abdominal crunches	TB protocol performed 6 d/wk consisting of 3–4 sets of 8–12 repetitions per exercise	- MRI: CSA of upper arm and thigh	8 wks
Villanueva et al. (40)	22 older, untrained men	Parallel group random assignment to 1 of 2 groups: (1) 60 s RI; (2) 240 s RI	45° bilateral leg press, flat bench machine chest press, lat pulldown, seated row, dumbbell step-ups, dumbbell Romanian deadlifts, bilateral knee extension/flexion	TB protocol performed 3 d/wk consisting of 2–3 sets of 4–6 repetitions per exercise	- DXA: FFM	8 wks

RI, rest interval; TB, total body; VL, volume load; FFM, fat-free mass; MT, muscle thickness; CIR, circumference; US, ultrasound; VM, vastus medialis; DXA, dual-energy x-ray absorptiometry; MRI, magnetic resonance imaging; BIA, bioelectrical impedance analysis.

### Meta-analysis of non-controlled effect sizes

**Figure 2:**
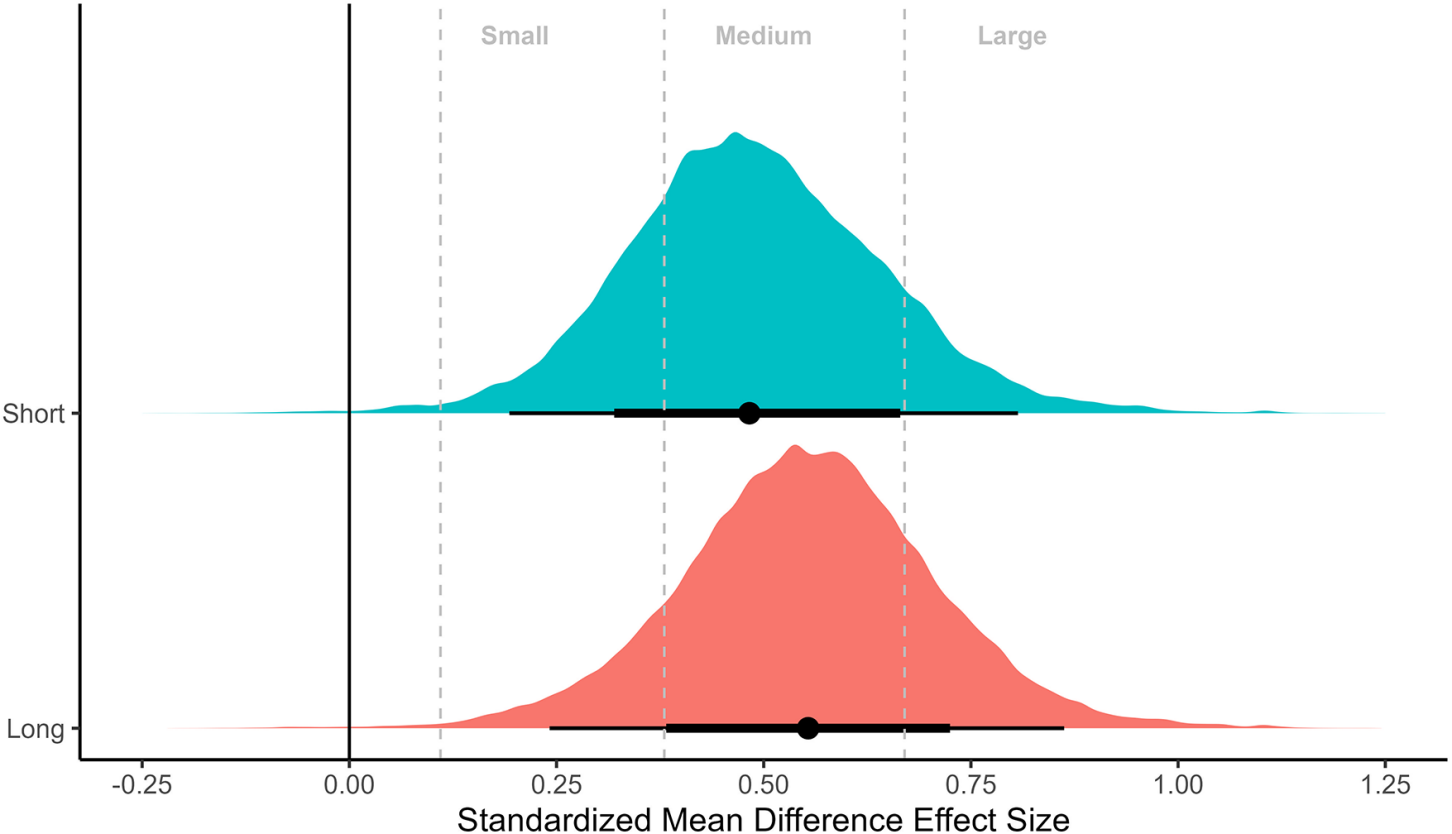
Meta-analysis of non-controlled effect sizes separated by binary categorization of short (≤60 s) vs. long (>60 s) inter-set rest periods. Plots illustrate shrunken posterior distribution of effect sizes following application of meta-analytic model. Circle: median, error bars represent 75 and 95% credible intervals. Small, medium, and large effect size thresholds are presented according to previous research in strength and conditioning (22).

**Figure 3:**
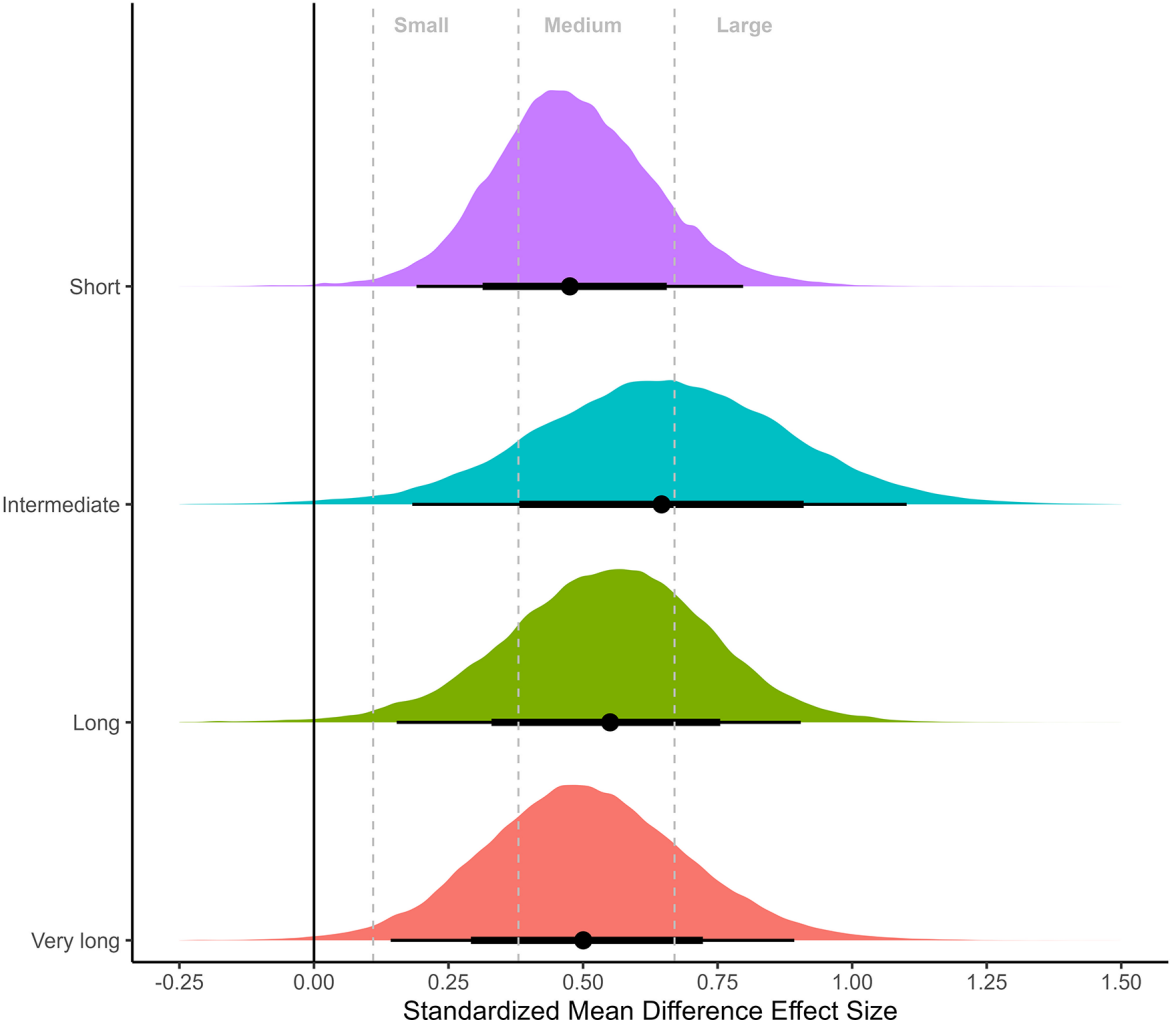
Meta-analysis of non-controlled effect sizes separated by short (≤60 s), intermediate (61 s–119 s), long (120–179 s), and very long (≥180 s) categorization of inter-set rest period. Plots illustrate shrunken posterior distribution of effect sizes following application of meta-analytic model. Circle: median, error bars represent 75 and 95% credible intervals. Small, medium, and large effect size thresholds are presented according to previous research in strength and conditioning (22).

Meta-analyses on non-controlled effect sizes using hierarchical models of all 19 measurements (thigh: 10; arm: 6; whole body: 3) from nine studies are presented in Figures 2, 3. Both meta-analyses showed substantial overlap of SMDs across the different inter-set rest periods [Binary: short: 0.48 (95%CrI: 0.19–0.81), longer: 0.56 (95%CrI: 0.24–0.86); Four categories: short: 0.47 (95%CrI: 0.19–0.80), intermediate: 0.65 (95%CrI: 0.18–1.1), long: 0.55 (95%CrI: 0.15–0.90), very long: 0.50 (95%CrI: 0.14–0.89)], with substantial heterogeneity in results. Central estimates suggested that improvements across the interventions were most likely to be between medium and large, highlighting that interventions included in this review were generally effective irrespective of rest interval duration.

### Meta-analysis of controlled effect sizes

**Figure 4:**
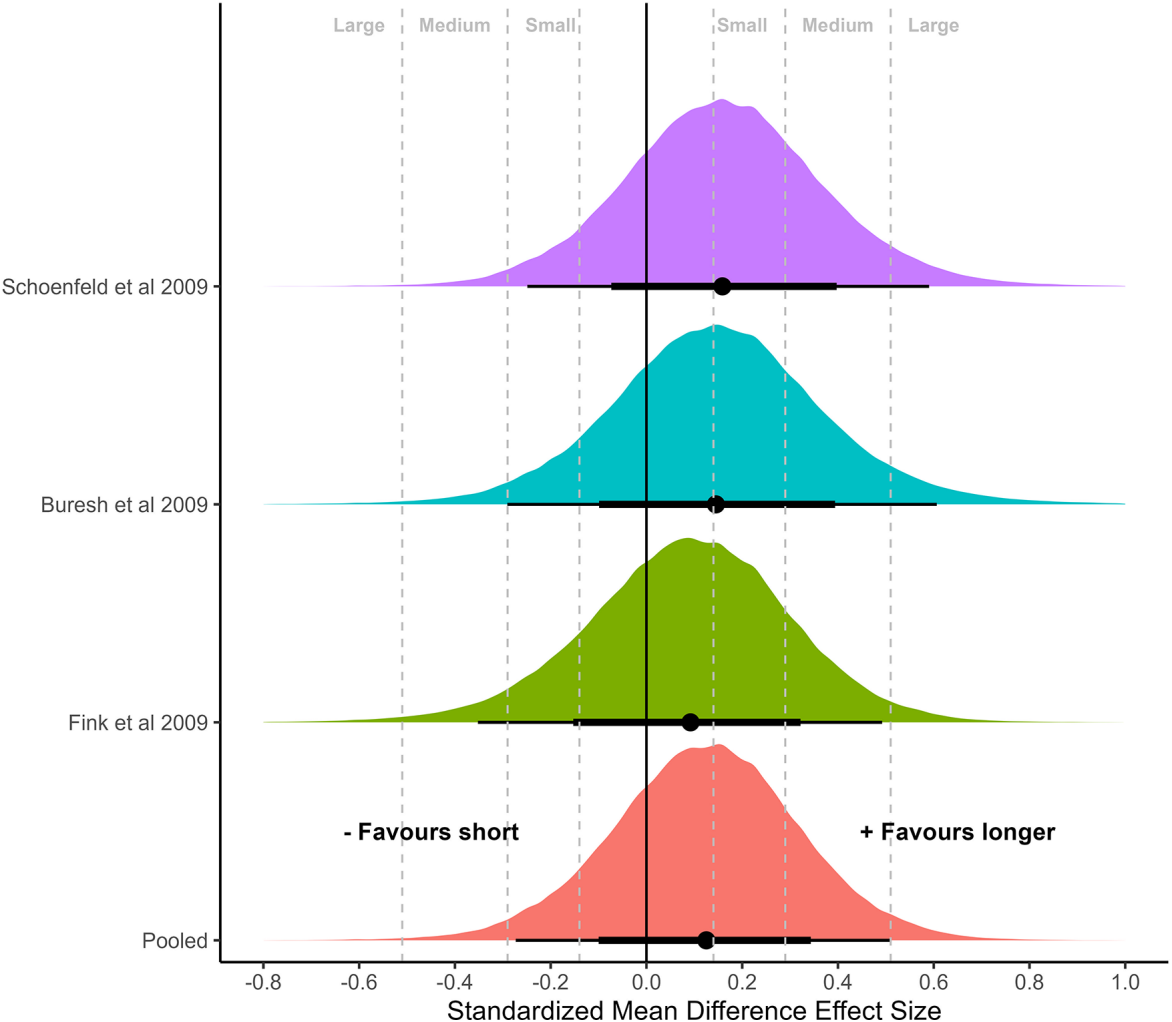
Meta-analysis of controlled effect sizes of muscular hypertrophy of the upper arm with direct comparisons of binary categorization of inter-set rest period. Plots illustrate shrunken posterior distribution of effect sizes following application of meta-analytic model. Circle: median, error bars represent 75 and 95% credible intervals. Small, medium, and large effect size thresholds are presented according to previous research in strength and conditioning (25). Probability of effect size greater than 0 favoring longer rest period = 0.74; probability of effect size greater than small favoring longer rest period = 0.45; probability of effect size greater than medium favoring longer rest period = 0.18; probability of effect size greater than large favoring longer rest period = 0.03.

**Figure 5:**
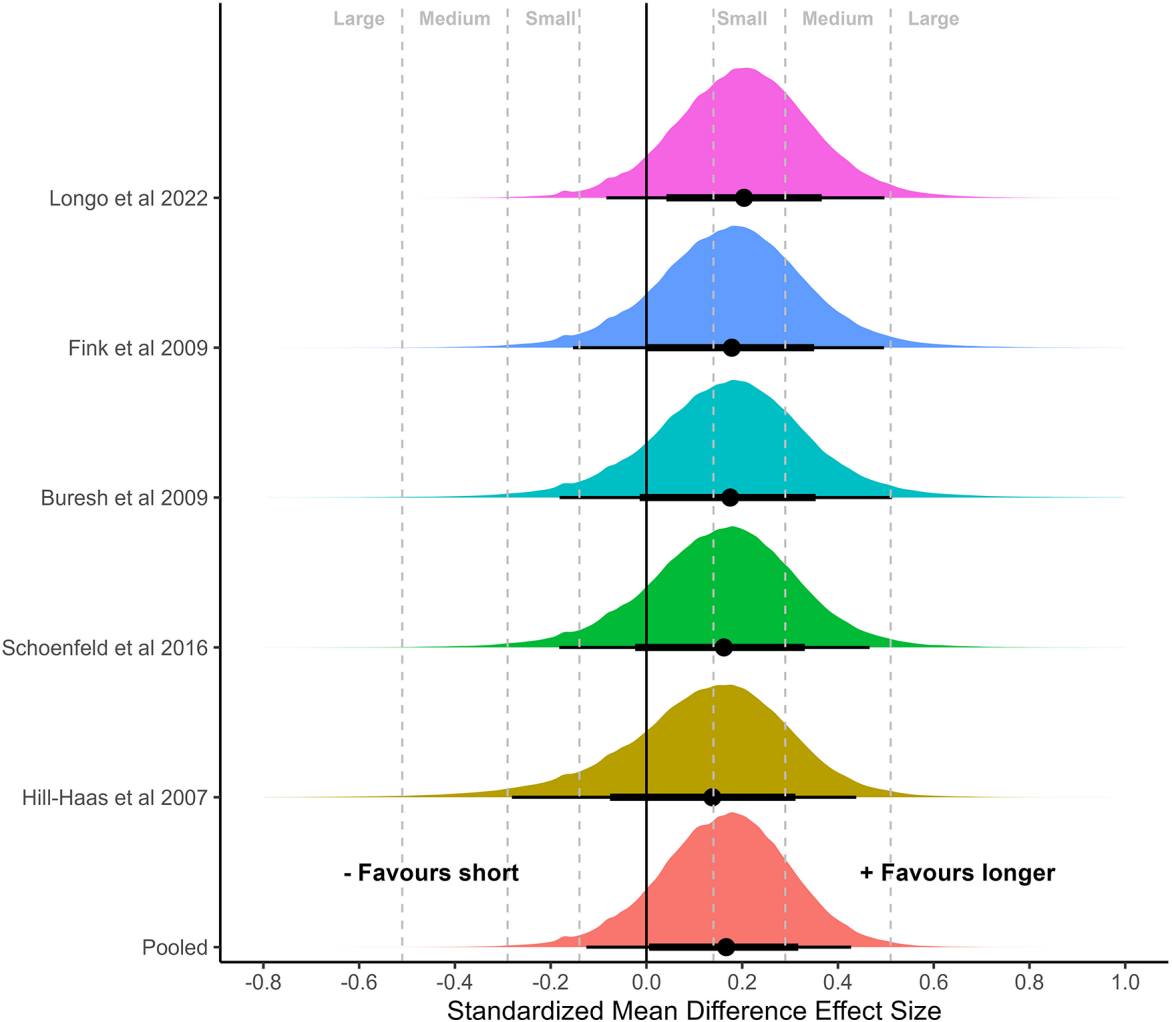
Meta-analysis of controlled effect sizes of muscular hypertrophy of the thigh with direct comparisons of binary categorization of inter-set rest period. Plots illustrate shrunken posterior distribution of effect sizes following application of meta-analytic model. Circle: median, error bars represent 75 and 95% credible intervals. Small, medium, and large effect size thresholds are presented according to previous research in strength and conditioning (25). Probability of effect size greater than 0 favoring longer rest period = 0.88; probability of effect size greater than small favoring longer rest period = 0.54; probability of effect size greater than medium favoring longer rest period = 0.15; probability of effect size greater than large favoring longer rest period = 0.01.

**Figure 6:**
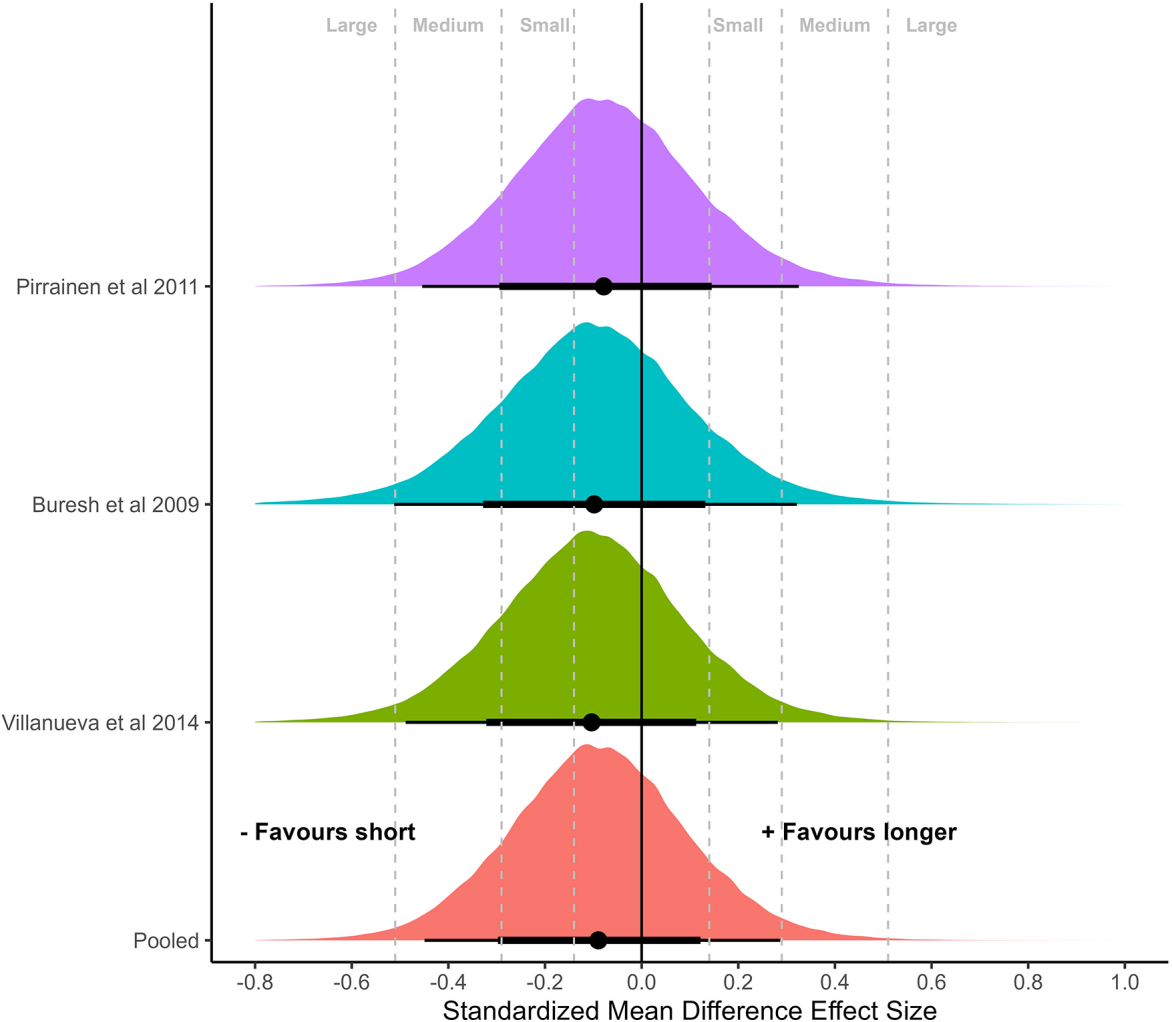
Meta-analysis of controlled effect sizes of muscular hypertrophy of the whole body with direct comparisons of binary categorization of inter-set rest period. Plots illustrate shrunken posterior distribution of effect sizes following application of meta-analytic model. Circle: median, error bars represent 75 and 95% credible intervals. Small, medium, and large effect size thresholds are presented according to previous research in strength and conditioning (25). Probability of effect size greater than 0 favoring short rest period = 0.69; probability of effect size greater than small favoring short rest period = 0.36; probability of effect size greater than medium favoring short rest period = 0.12; probability of effect size greater than large favoring short rest period = 0.01.

Univariate and multivariate meta-analyses of controlled binary (short vs. longer) effect sizes were conducted for outcomes separated by body region (arm, thigh, whole body; Figures 4–6). Similar results were obtained for the arm and thigh with central estimates slightly favoring longer rest periods [arm: 0.13 (95%CrI: −0.27 to 0.51); τ: 0.10 (75%CrI: 0.02–0.31), Figure 4; thigh: 0.17 (95%CrI: −0.13 to 0.43); τ: 0.17 (75%CrI: 0.02–0.22), Figure 5]. In contrast, central estimates closer to zero but slightly favoring shorter rest periods were estimated for the whole body [whole body: −0.08 (95%CrI: −0.45 to 0.29); τ: 0.08 (75%CrI: 0.02–0.27), Figure 6]. Application of the multivariate meta-analysis model resulted in slight reductions in uncertainty with smaller central estimates all modestly favoring longer rest periods [arm: 0.11 (95%CrI: −0.26 to 0.48); thigh: 0.16 (95%CrI: −0.13 to 0.41); whole body: 0.03 (95%CrI: −0.28 to 0.36)].

Controlled effect sizes for the four categories of inter-set rest period were analyzed with network meta-analyses. Sufficient data were available for univariate analysis of the arm and thigh. Network structures are presented in the supplementary files, with effect size estimates combining direct and indirect estimates, and SUCRA values presented in Table 2. In general, effect size estimates and SUCRA values for both regions of the body indicated greater effectiveness for rest periods beyond the short categorization. In general, effect size estimates and SUCRA values ranking rest periods indicated greater effectiveness for durations beyond the short categorization in both regions of the body.

**Table 2:** Univariate network meta-analyses combining direct and indirect pairwise comparisons for hypertrophy at the thigh and arm for the four inter-set rest period categories.

Region	Category	Comparative effect size (95%CrI)	SUCRA
Arm	Short	–	0.40
	Intermediate	0.22 (−0.31 to 0.74)	0.49
	Long	−0.02 (−0.43 to 0.37)	0.52
	Very long	0.18 (−0.36 to 0.70)	0.60
Thigh	Short	–	0.18
	Intermediate	0.13 (−0.31 to 0.58)	0.54
	Long	0.01 (−0.39 to 0.41)	0.63
	Very long	0.32 (−0.10 to 0.68)	0.64

Comparative effect sizes are expressed relative to the short inter-set rest category. CrI, Credible interval; SUCRA, Surface Under the Cumulative Ranking curve.

### Subanalyses

Subanalyses were performed on direct comparisons of binary effect sizes separating studies based on set end-point (i.e., training to momentary muscular failure or non-failure) and training status (specific to designs that included untrained participants). A multivariate analysis comprised of data from three studies that incorporated training to momentary muscular failure was conducted for hypertrophy of the thigh [0.31 (95%CrI: −0.03 to 0.61)] and arm [0.04 (95%CrI: −0.37 to 0.44)]. Similarly, a multivariate analysis comprised of data from three studies that incorporated non-failure RT was conducted for hypertrophy of the thigh [0.27 (95%CrI: −0.02 to 0.51)] arm [0.04 (95%CrI: −0.37 to 0.44)], and whole body [−0.06 (−0.40 to 0.27)]. Consistency in results provided no evidence of a difference in the influence of rest periods for different set end-points. Finally, sufficient data were available to perform a multivariate analysis comprised of data from six studies that included untrained participants and was conducted for hypertrophy of the thigh [0.17 (95%CrI: −0.15 to 0.47)] arm [0.02 (95%CrI: −0.41 to 0.46)], and whole body [−0.05 (−0.43 to 0.26)]. Insufficient data were available to subanalyze results in trained individuals.

Below is a funnel plot that illustrates calculated effect sizes from binary categorisation (shorter versuslonger rest periods) for muscular hypertrophy measured at the arms (upper), thighs (lower) and whole body. Data points are clustered around the central pooled estimate (vertical line) and its 95% credible interval (rectangular shaded region). Plot illustrates no concern with small-study effects.

### Analyses of small study bias

Visual inspection of the funnel plot indicates no evidence of small study bias (see supplemental file).

### Methodological qualitative assessment

Qualitative assessment of included studies via the SMART-LD tool showed a mean score of 15 out of a possible 20 points (range: 12–17 points). Four studies were judged to be of good quality (34, 37, 38, 40), 4 studies were judged to be of fair quality (32, 35, 36, 39), and 1 study was judged to be of poor quality (33), see supplementary files.

## Discussion

Our meta-analysis quantified data from studies that directly compared the effects of different rest interval lengths on measures of muscle hypertrophy. While the initial meta-regressions with non-controlled effect sizes highlighted substantial heterogeneity across studies (Figures 2, 3), they also demonstrated that most interventions were effective in eliciting hypertrophic adaptations regardless of rest interval duration, with SMDs that could be considered medium to large in magnitude. Binary categorization comparing short (≤60 s) with longer (>60 s) rest intervals returned slightly greater central estimates favoring the longer rest condition (SMD = 0.56 vs. 0.48, respectively; Figure 2). When further stratifying data, results showed slight differences between short (SMD = 0.47), intermediate (SMD = 0.65), long (SMD = 0.55) and very long (SMD = 0.50) rest periods (Figure 3). These results suggest no clear benefit to altering rest interval length for the purpose of promoting muscle hypertrophy. However, given substantial heterogeneity, meta-regressions with a small number of studies provide limited ability to draw strong inferences as any differences observed can be the result of chance imbalances in the distribution of studies. Therefore, the primary inference from this study was focused on meta-analyses that comprised controlled effect sizes with either direct pairwise comparisons only (bivariate categorization), or both direct and indirect pairwise comparisons (four categories) through network models.

Meta-analyses were conducted within a Bayesian framework as is most common with network models to naturally produce ranking and probability outputs to better interpret results (41). Additionally, Bayesian models allow for the use of informative priors which were placed here on the sampling error of effect sizes, the between study variation, and the effect size values using previous knowledge to enhance precision of estimates.

### Sub-analysis of body regions

When subanalyzing the effects of rest interval length on hypertrophy of the upper and lower limbs, the results suggest a small benefit for rest intervals >60 s. For the binary categorization, the pooled effect size for the arms slightly favored a hypertrophic benefit for longer vs. shorter rest durations (SMD = 0.13). The probability of the effect being greater than zero was 74%, with only a 45% probability that the difference in effect was greater than small. Similarly, the pooled effect size for quadriceps femoris modestly favored longer vs. shorter durations (SMD = 0.17). There was a strong probability that this effect was greater than zero (88%), but only a 54% probability that the difference in effect was greater than small. Both upper and lower limb analyses showed a very low probability that differences would be greater than a medium effect (SMD = 0.18 and 0.15, respectively). Conversely, measures of whole-body hypertrophy showed slightly greater effects favoring shorter vs. longer rest durations (SMD = −0.08, *p* (>0) = 0.69, *p* (>small) = 0.36); however, with substantial uncertainty due to only three studies providing whole body data.

Potential discrepancies between findings of hypertrophy of the extremities vs. the whole body may be related to the different methods of assessment. Whole-body measures of muscle growth were based on estimates of fat-free mass (FFM) via DXA, BIA and hydrodensitometry, which are often used as proxies for muscle hypertrophy (42). However, FFM encompasses all bodily tissues other than fat mass; while alterations in skeletal muscle comprise the majority of FFM changes that occur during RT, other components such as water and mineral can influence results as well (43). Alternatively, the majority of assessments for the extremities employed direct measurements of changes in muscle mass via MRI and ultrasonography. Given that direct assessment methods have been shown to be more sensitive to detecting RT-induced hypertrophy than indirect assessments (44, 45), the results of our whole-body analysis should be interpreted with caution.

### Rest interval duration and volume load

Potential beneficial effects of rest periods greater than 60 s on muscle hypertrophy may be attributable to preservation of volume load during a training session. Research indicates that short rest periods (≤60 s) appreciably reduce the number of repetitions performed across multiple sets compared to longer rest durations (11, 12, 46), which could have a detrimental effect on long-term muscular adaptations. This hypothesis is supported by Longo et al. (35), who reported appreciably greater increases in quadriceps femoris cross-sectional area when training with 180 vs. 60 inter-set rest periods over a 10-week intervention (13.1% vs. 6.8%, respectively); of note, volume load was reduced to a significantly greater extent in the shorter vs. longer rest condition (average number of repetitions across 3 sets: 9.8 ± 2.9 vs. 16.1 ± 5.2, respectively). However, similar hypertrophy was observed with the performance of additional sets to equate volume load between conditions.

Alternatively, previous evidence suggests that differences in volume load tend to level off when comparing rest intervals of 120 vs. 180 s (11, 46). When compared to very short rest intervals (≤60 s), our network meta-analysis suggested that very long rest intervals (≥180 s) provided a modest advantage vs. intermediate (61–119 s) and long (120–179 s) durations with respect to quadriceps femoris hypertrophy. However, these data showed a high degree of uncertainty and the U-shaped response in the median estimates between conditions casts further doubt on the veracity of the finding. Analyses of arm hypertrophy did not show an appreciable effect of rest interval durations beyond intermediate (>60 s) durations. Future research should explore this topic in greater detail to better determine whether graded increases in rest interval durations alter muscular adaptations as well as the extent to which volume load may play a role in the process.

### Sub-analysis of proximity-to-failure

Subanalysis of set end-point found that the proximity-to-failure of set termination (i.e., failure or non-failure) did not meaningfully influence the interaction between rest interval duration and muscle hypertrophy. Central estimates from both analyses suggested a hypertrophic benefit for longer rest periods in the quadriceps femoris, irrespective of the proximity-to-failure reached during RT. However, the magnitude of effect was relatively small (SMD = 0.27 and 0.31 for non-failure and failure conditions, respectively). Alternatively, negligible differences were observed for the influence of rest interval length in the arms (SMD = 0.04) regardless of proximity-to-failure. The findings are somewhat in contrast with data showing that shorter rest periods impair bench press performance to a greater extent than longer rest periods when training with closer proximities to failure (47). Further research is needed to better understand the potential discrepancies between acute and longitudinal outcomes.

### Sub-analysis of participant training status

Subanalysis of the potential influence of training status on rest interval length showed that untrained individuals displayed a slight hypertrophic benefit from longer rest periods when training the quadriceps femoris (SMD = 0.17). However, rest interval length appeared to have negligible effects on measures of arm and whole-body hypertrophy in untrained individuals (SMD = 0.02 and −0.05, respectively). These data are relatively consistent with findings from a systematic review by Grgic et al. (15) that concluded both shorter and longer rest durations are equally viable options for promoting hypertrophy in novice trainees. The systematic review by Grgic et al. (15) also suggested that trained individuals might benefit from the use of longer rest intervals, conceivably by allowing for a greater volume load across multi-set protocols. Unfortunately, there was insufficient data to subanalyze results on trained lifters, precluding our ability to further generalize this claim. Further research is therefore needed to better understand how training status may influence the response to rest interval length.

### Limitations

Our analysis has several limitations that should be considered when drawing practical inferences for exercise prescription. First, the included studies had substantial heterogeneity in exercise selection, with the protocols employing varying use of free weights and machines as well single-joint and multi-joint movements (and, in some cases, combinations of these modes). Given that the complexity of an exercise may alter the fatigue response across sets (11), it is conceivable that rest interval prescription should vary based on the type of exercise employed. Second, no studies have investigated the effect of rest interval length on the muscles of the torso (i.e., pectorals, latissimus dorsi, deltoids etc); it is possible that these muscle groups may respond differently to shorter rest durations than those of the limbs, although this seems unlikely. Third, the volume of training was generally moderate for the included studies; therefore, it remains undetermined how differences in rest interval length might influence hypertrophy with a higher number of sets performed per muscle group. Fourth, the majority of studies to date have been carried out on untrained, younger participants. Further study is therefore warranted in resistance-trained individuals and older adults to better generalize findings to this population. Finally, while the observed differences in effect are likely to be between zero and small, intervention durations were relatively short (between 5 and 10 weeks); thus, it is possible that accumulated differences in muscle mass accretion may be more appreciable over longer time frames.

## Conclusion

This meta-analysis indicates that hypertrophy can be achieved across a wide spectrum of rest interval ranges but suggests a small benefit to employing longer vs. shorter inter-set rest intervals for muscle hypertrophy. The effect favoring longer inter-set rest intervals was relatively consistent between the arms and the legs musculature, and results were not meaningfully influenced by whether RT was performed to failure or non-failure. These findings are inconsistent with recommendations from the National Strength and Conditioning Association, which prescribe relatively short rest periods (30–90 s) for hypertrophy-related goals (7). Thus, current guidelines regarding rest interval prescription for achieving muscular hypertrophy warrant reconsideration.

The current evidence remains equivocal as to whether resting more than 90 s between sets further enhances hypertrophic adaptations. Our analysis casts doubt as to any beneficial effects in this regard. However, given the uncertainty of evidence, additional studies are needed comparing measures of hypertrophy across a wide spectrum of rest periods to provide better insights on the topic.

## Data Availability

Data and supplementary material are available on the Open Science Framework project page: https://osf.io/zp6vs/.

## Footnotes

^1^In cases where studies equated sets between conditions, fewer repetitions may have been performed in the shorter rest conditions over multiple sets of a given exercise.
